# Network analysis exposes core functions in major lifestyles of fungal and oomycete plant pathogens

**DOI:** 10.1186/s12864-019-6409-3

**Published:** 2019-12-26

**Authors:** Eswari PJ Pandaranayaka, Omer Frenkel, Yigal Elad, Dov Prusky, Arye Harel

**Affiliations:** 1 Department of Vegetable and Field Crops, Institute of Plant Sciences, Volcani Center, Agricultural Research Organization, Rishon LeZion, Israel; 2 Department of Plant Pathology and Weed Research, Institute of Plant Protection, Volcani Center, Agricultural Research Organization, Rishon LeZion, Israel; 3 Department of Postharvest Science, Institute of Postharvest and Food Sciences, Volcani Center, Agricultural Research Organization, Rishon LeZion, Israel

**Keywords:** Fungus–plant interaction, Network, Core function, Plant pathogen, Necrotroph, Biotroph, Hemibiotroph, Virulence, Comparative genomics

## Abstract

**Background:**

Genomic studies demonstrate that components of virulence mechanisms in filamentous eukaryotic pathogens (FEPs, fungi and oomycetes) of plants are often highly conserved, or found in gene families that include secreted hydrolytic enzymes (e.g., cellulases and proteases) and secondary metabolites (e.g., toxins), central to the pathogenicity process. However, very few large-scale genomic comparisons have utilized complete proteomes from dozens of FEPs to reveal lifestyle-associated virulence mechanisms. Providing a powerful means for exploration, and the discovery of trends in large-scale datasets, network analysis has been used to identify core functions of the primordial cyanobacteria, and ancient evolutionary signatures in oxidoreductases.

**Results:**

We used a sequence-similarity network to study components of virulence mechanisms of major pathogenic lifestyles (necrotroph (ic), N; biotroph (ic), B; hemibiotroph (ic), H) in complete pan-proteomes of 65 FEPs and 17 saprobes. Our comparative analysis highlights approximately 190 core functions found in 70% of the genomes of these pathogenic lifestyles. Core functions were found mainly in: transport (in H, N, B cores); carbohydrate metabolism, secondary metabolite synthesis, and protease (H and N cores); nucleic acid metabolism and signal transduction (B core); and amino acid metabolism (H core). Taken together, the necrotrophic core contains functions such as cell wall-associated degrading enzymes, toxin metabolism, and transport, which are likely to support their lifestyle of killing prior to feeding. The biotrophic stealth growth on living tissues is potentially controlled by a core of regulatory functions, such as: small G-protein family of GTPases, RNA modification, and cryptochrome-based light sensing. Regulatory mechanisms found in the hemibiotrophic core contain light- and CO_2_-sensing functions that could mediate important roles of this group, such as transition between lifestyles.

**Conclusions:**

The selected set of enriched core functions identified in our work can facilitate future studies aimed at controlling FEPs. One interesting example would be to facilitate the identification of the pathogenic potential of samples analyzed by metagenomics. Finally, our analysis offers potential evolutionary scenarios, suggesting that an early-branching saprobe (identified in previous studies) has probably evolved a necrotrophic lifestyle as illustrated by the highest number of shared gene families between saprobes and necrotrophs.

## Background

Filamentous eukaryotic pathogens (FEPs; i.e., fungi and oomycetes) of plants cause extensive losses in annual yields of staple crops worldwide [1, 2]. The danger posed by these pathogens is enhanced by accelerated pathogen evolution, mainly due to the continuous use of fungicides in monoculture practice, and human- or climate-dependent dispersal [2, 3]. Understanding the genetic basis of fungal and oomycete pathogenicity mechanisms may provide new avenues for the development of revamped disease-control strategies. Despite the increasing number of sequenced FEP genomes (e.g., the 1000 fungal genomes from the Joint Genome Institute (JGI) [4]), there are very few large-scale genomic comparisons that make use of complete proteomes from at least a few dozen FEP genomes, which could reveal novel and niche-specific virulence mechanisms (e.g., study of proteases in [5, 6], and cell wall-degrading enzymes in [7]).

Genomic studies have shown that components of virulence mechanisms in FEPs are often highly conserved, or found in gene families that are potentially generated due to their association with the high genomic plasticity found in many of these pathogens [8–15]. One example is the conserved signaling module in fungi and oomycetes (i.e., the phosphorylative regulation machinery), which is pivotal for sensing environmental cues, and for regulating infection-associated morphogenetic transitions in pathogens [13, 16–19]. Comparative genomic studies have also pinpointed the dispersal of conserved effector families and domains across FEP species: (i) LysM domain-containing effectors that sequester chitin oligosaccharides from host defense [20]; (ii) toxins (TOXB, TOX2, HC, and Nep1-like proteins) [21–23]; (iii) the RXLR sequence motif mediating host translocation in oomycete effectors [24]; (iv) CRN effectors, cell death-inducing oomycete effectors [8]; and (v) Hce2s effectors potentially involved in adaptation to stress [25]. In addition, the capacity to generate and coordinately secrete proteins and secondary metabolites is prevalent in these pathogens, and central to their pathogenicity process [26]. These secreted components include a large arsenal of hydrolytic enzymes (e.g., cellulases, pectinases, proteases, lipases), oxidoreductases [27–29], and metabolites (e.g., polyketides, terpenes, and nonribosomal peptide (NPS)) effectors, some of them diverse, and tailored to a specific host [21, 24, 30]. Despite their high diversity and host specificity, over half of the predicted effectors are part of gene families- in 3 studied species of *Pucciniomycetes* (51 to 68% of the effectors), 2 *Phytophthora* species (77% of the effectors), and 18 *Dothideomycetes* (79% of the total count of effectors from all 18 species) [9, 31–33]. The correlation of certain gene families to specific lifestyles has facilitated defining metabolic activity, and the pathogenicity mechanisms required for different ecological niches [9, 33].

Three major lifestyles are known in fungal and oomycete phytopathogens. The necrotrophic lifestyle (hereafter, N is used to refer to necrotrophs), which is characterized by killing of the host cell before feeding on its dead tissue, is involved in utilizing host-selective toxin effectors (e.g., ToxA, Tox1/2/4, and Nep1-like proteins) (in) directly interacting with a host-susceptibility gene product, and ultimately leading to cell death [21, 24, 34, 35]. One example in this category is the broad host range fungus *Botrytis cinerea*, capable of infecting over 1400 plant species (including 200 cultivars) [36]. The biotrophic lifestyle (B will refer to biotrophs), which is characterized by nutrition and growth on living tissue, requires avoidance of plant defense mechanisms while feeding on the host compounds. One example in this category is *Erysiphe necator*, known to cause powdery mildew in grapes [37]. The hemibiotrophic lifestyle (H will refer to hemibiotrophs) is characterized by an initial biotrophic infection mode, followed by a transition to the necrotrophic stage. One example in this category is the fungus *Colletotrichum gloeosporioides* which causes significant damage to subtropical and tropical fruit before and after harvest [38]. In contrast to the pathogenic lifestyles, the saprotrophic lifestyle (Sap) is characterized by nutrition and growth on organic matter or decaying tissue [39]. One example in this category, is the model filamentous fungus *Neurospora crassa* [40]. Processing of organic/decaying tissue is typically associated with extracellular enzymatic degradation and subsequent absorption of nutrients. A fundamental aspect of the plant–pathogen interaction is induction of plant defense as a result of recognition of often conserved pathogen-associated molecular patterns (PAMPs, e.g., glucans, and chitin) by pathogen recognition receptors (PRRs) [24, 41–45], which is often termed PAMP-triggered immunity (PTI). Pathogens secrete effectors which suppress this primary defense mechanism (i.e., the PTI) and allow them to infect plants [24, 41–45]. In turn, plants evolved to produce R proteins (mainly nucleotide binding–leucine-rich repeat (NB-LRR) receptors) which invoke the plant defense upon (in) direct recognition of pathogen effectors, termed effector-triggered immunity (ETI) [43–45]. The effectors participate in both the (hemi) biotrophic and necrotrophic virulence processes, and their activity is important for avoidance of plant defense mechanisms in biotrophs.

To the best of our knowledge, there are very few large-scale genomic comparisons that make use of complete proteomes from dozens of FEP genomes, to discover novel, and niche-specific virulence mechanisms (e.g., study of proteases in [5, 6], and cell wall-degrading enzymes in [7]). The power of such analyses was demonstrated in the study of 18 *Dothideomycetes* genomes with diverse lifestyles (3 Sap, 6 N, 2 B, and 7 H) compared to outgroup genomes. That study identified 3 K core gene families (comprised of 66 K genes) of *Dothideomycetes* having at least one representative in each *Dothideomycetes* genome, containing 233 Pfam domains that were expanded in *Dothideomycetes* compared to the control. These core gene families also contained 69 Pfam domains that were expanded in *Dothideomycetes* pathogens vs. outgroup pathogens [33]. Empowered by diverse multiple genomes of *Dothideomycetes*, that analysis highlighted gene families potentially playing a role in necrotrophic, hemibiotrophic, and saprotrophic lifestyles, primarily within the *Dothideomycetes* class of Ascomycota. Following observation of the conserved pathogenicity mechanisms mentioned above, and common characteristics of the major pathogenic lifestyles, we hypothesize that it is feasible to deploy the power of comparative genomics analysis in a large set of FEPs to identify core functions of pathogenicity for those lifestyles, as partially demonstrated in the *Dothideomycetes* class.

Networks offer a fashionable methodology for studying large-scale multifaceted genomic and functional genomic data [46]. Enabling integration of metadata [47, 48], it can facilitate the correlation of genomic elements and pathways with diverse pathogenic lifestyles (e.g., (hemi) biotrophic, and necrotrophic). Supported by a mathematical background for analysis and validation of the results, it provides a powerful means for exploration, and the discovery of trends in large-scale datasets, such as multiple genomes of FEPs. In our current study, we used sequence-similarity network analysis [46, 48] encompassing available complete pan-proteomes of 82 fungi and oomycetes (18 B, 20 H, 22 N, and 17 Sap; Additional file 2: Table S1) to identify components of virulence mechanisms. Our comparative analysis highlights approximately 190 significantly enriched core functions found in 70% of the genomes of a pathogenic lifestyle (e.g., core necrotrophic functions are shared by 70% of the genomes in this lifestyle). This includes functions that are specifically enriched in one lifestyle, and functions that are shared between pathogenic lifestyles. We show that these core functions can assist in discriminating the different pathogenic lifestyles. Finally, empowered by network analysis, our study of shared families in the entire set of 82 genomes illustrates potential evolutionary routes between these lifestyles.

## Results

**Fig. 1:**
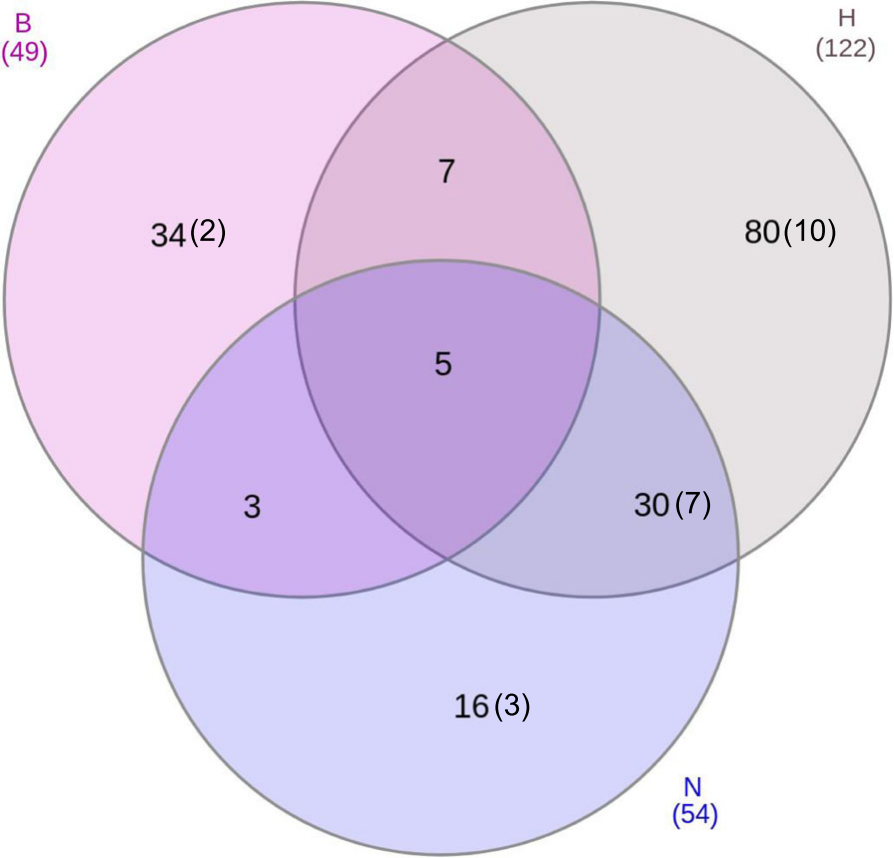
Distribution of significantly enriched unique functions (annotation IDs) among the pathogenic lifestyles. B – biotroph, H – hemibiotroph, N – necrotroph. Number in parentheses indicate counts of significantly enriched functions containing SSPs which include cutin and pectin degradation, cutinase, secondary metabolism, proteinaceous toxins, glycoside hydrolase, and signal transduction (tyrosine phosphatase activity) in the HN cores

Our pan-proteome network consisted of approximately 3.9 K core gene families shared by at least 70% of a lifestyle (see section “Core components”, Methods). Approximately 40% of these core families were shared among all four lifestyles, i.e., core of all lifestyles (center of the Venn diagram, Additional file 1: Figure S1), and 25% were unique core families of only one lifestyle. The highest number of cores was found in H, followed by N, and B (Additional file 1: Figure S1). Hereafter, Ncore refers to the core of N (Bcore to core of B, Hcore to core of H, and Sapcore to core of Sap). Most of the proteins (≥89%) in the core gene families were annotated for having either KEGG orthologs, InterPro domains or MEROPS proteases (Additional file 2: Table S2). Based on these annotations, we identified approximately 190 significantly enriched functions (see section “Calculation of enrichment and significance of core pathogenic functions”, Methods) in the core gene families of lifestyles H, B, and N (Fig. 1). All downstream analyses, unless otherwise specified, were based on these functions (often referred to as core functions). These core functions consisted of annotations which were enriched in only one lifestyle (i.e., B, H, or N), and annotations shared between several pathogenic lifestyles (Fig. 1). Around 4% of the core families did not contain proteins with the above-specified annotations, and only 6% of these unannotated families contained small secreted proteins (SSPs).

### Core gene families may assist in discriminating between the pathogenic lifestyles

**Fig. 2:**
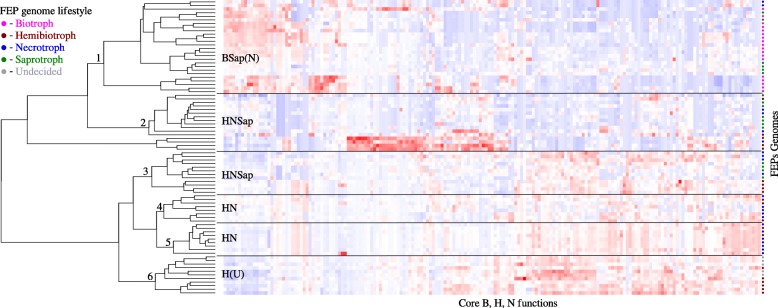
Hierarchical clustering of the 65 selected FEP and 17 saprophyte genomes based on significantly enriched core functions. X-axis represents core functions (Additional file 2: Table S3, Methods), and Y-axis represents studied genomes (Additional file 2: Table S1, Methods). Six major clusters are indicated by numbers above the tree branches (left). B – biotroph, H – hemibiotroph, N – necrotroph, Sap – saprotroph. Lifestyle of each of the FEP genomes is indicated by filled circles (Y-axis, see color code, top left)

To test whether the identified core functions can be used to differentiate between pathogenic lifestyles, we utilized hierarchical clustering (Fig. 2). The clustering analysis showed separation of B genomes from other pathogenic lifestyles (with the exception of 2 N genomes; see cluster 1 in Fig. 2). N and H genomes appeared in 5 clusters (clusters 2–6 in Fig. 2): clusters 2 and 3 also contained Sap, while clusters 4–6, which contained most (55%) of the N and H genomes, did not contain Sap. Cluster 6 contained only H, along with all of the ambiguously characterized genomes (indicated by U, undecided, Fig. 2). The shared clustering of H and N corresponded with the highest number of shared functions within this lifestyle pair (HN column, Table 1).

**Table 1:** Frequency of annotation IDs that are significantly enriched in core components of pathogenic lifestyles within selected functional categories (see section “Calculation of enrichment and significance of core pathogenic functions”, Methods). Number in parentheses indicates percentage of annotations in a lifestyle, e.g., there are 10 annotations related to nucleic acids in B which represent 29.4% of the annotations of B. Numbers in bold represent abundant fuctional categories. Detailed annotations are illustrated in Additional file 2: Table S3

Functional category/Core ^a^	B (%) ^b^	H (%)	N (%)	HN (%)	BH (%)	NB (%)	BHN (%)
**Amino acid metabolism**	2 (5.9)	**12 (15.0)**	1 (6.3)	0 (0.0)	0 (0.0)	0 (0.0)	0 (0.0)
**Carbohydrate metabolism**	2 (5.9)	**22 (27.5)**	1 (6.3)	**11 (36.7)**	1 (14.3)	0 (0.0)	0 (0.0)
**Nucleic acid related**	**10 (29.4)**	4 (5.0)	0 (0.0)	2 (6.7)	4 (57.1)	1 (33.3)	0 (0.0)
**Protease**	3 (8.8)	5 (6.3)	3 (18.8)	**5 (16.7)**	0 (0.0)	1 (33.3)	4 (80.0)
**Secondary metabolites**	3 (8.8)	**19 (23.8)**	2 (12.5)	**7 (23.3)**	0 (0.0)	0 (0.0)	1 (20.0)
**Signal transduction**	**6 (17.7)**	3 (3.8)	0 (0.0)	0 (0.0)	0 (0.0)	0 (0.0)	0 (0.0)
**Transporters**	**6 (17.7)**	**16 (20.0)**	**7 (43.8)**	**6 (20.0)**	2 (28.6)	0 (0.0)	0 (0.0)
Chaperone	0 (0.0)	1 (1.3)	0 (0.0)	0 (0.0)	0 (0.0)	0 (0.0)	0 (0.0)
CO_2_ sensing	0 (0.0)	1 (1.3)	0 (0.0)	0 (0.0)	0 (0.0)	0 (0.0)	0 (0.0)
Energy	1 (2.9)	1 (1.3)	0 (0.0)	1 (3.3)	0 (0.0)	0 (0.0)	0 (0.0)
Light sensing and light-responsive nucleic acid functions	1 (2.9)	1 (1.3)	0 (0.0)	0 (0.0)	4 (57.1)	0 (0.0)	0 (0.0)
Oxidoreductases	0 (0.0)	2 (2.5)	1 (6.3)	1 (3.3)	0 (0.0)	0 (0.0)	0 (0.0)
Symbiosis	0 (0.0)	0 (0.0)	0 (0.0)	0 (0.0)	0 (0.0)	1 (33.3)	0 (0.0)
Trafficking	3 (8.8)	7 (8.8)	0 (0.0)	1 (3.3)	0 (0.0)	1 (33.3)	0 (0.0)
Translation	3 (8.8)	1 (1.3)	0 (0.0)	0 (0.0)	0 (0.0)	0 (0.0)	0 (0.0)
Unknown	1 (2.9)	4 (5.0)	2 (12.5)	0 (0.0)	0 (0.0)	0 (0.0)	0 (0.0)
Vitamin	0 (0.0)	2 (2.5)	0 (0.0)	1 (3.3)	0 (0.0)	0 (0.0)	0 (0.0)
Total annotation ID count^c^	34	80	16	30	7	3	5

^a^ *B* biotroph, *H* hemibiotroph, *N* necrotroph, *HN* hemibiotroph and necrotroph, *BH* biotroph and hemibiotroph, *NB* necrotroph and biotroph, *BHN* biotroph, hemibiotroph, and necrotroph

^**b**^ Counts of annotations (e.g., KEGG ortholog ID) associated with a function

^c^ One annotation ID is counted only once even if it occurs in multiple functions

### Core gene families of pathogenic lifestyles

In the analysis of core functions, most were found to belong to 7 abundant functional categories (bold in Table 1) which contained at least 10% of the annotations of a pathogenic lifestyle, and at least 5 significantly enriched annotations. These abundant functional categories included: transport in H, N, and Bcores; carbohydrate metabolism, secondary metabolite synthesis, and protease in H and Ncores; nucleic acid metabolism, and signal transduction in Bcores; and amino acid metabolism in Hcores. Other less abundant functional categories that contained significantly enriched annotations in B, H, and Ncores included trafficking, light-mediated functions, signal transduction, uncharacterized oxidoreductases, CO_2_ sensing, and chaperones. In addition, we identified several significantly enriched uncharacterized domains or KEGG orthologs in the cores of each of the pathogenic lifestyles (designated as unknown in Table 1 and Additional file 2: Table S3). Some abundant functional categories (bold in Table 1) characterized only one pathogenic lifestyle (e.g., amino acid metabolism in Hcores), whereas others were abundant in more than one lifestyle (e.g., transport). Hereafter, functions shared by more than one lifestyle are referred to as lifestyle1lifestyle2cores; e.g., HNcores which contain functions enriched in both H and N cores.

### Core gene families shared between pathogenic lifestyles

HNcores contained significantly enriched functions abundant in (Table 1, and corresponding detailed annotations in Additional file 2: Table S3): (i) carbohydrate metabolism related to cell wall-associated (i.e., including the cuticle) degradation and remodeling, such as pectinase, cutinase, and glycoside hydrolase family 28; (ii) secondary metabolite synthesis related mainly to toxins, and xenobiotic compound degradation and toxin synthesis; (iii) transport related to toxins and phospholipids; (iv) proteases related to serine peptidases of families 8–10, and metallopeptidase family M28. BHcores were significantly enriched in cryptochrome/photolyase-based DNA-repair functions, and in less abundant functions, such as transporters of glycerol, urea, and CO_2_; and glucanases (carbohydrate metabolism). Our analysis also identified a few functions that were significantly enriched in the cores of all three pathogenic lifestyles (BHNcores), such as members of serine peptidase family 8, and acyl-CoA oxidase participating in protein kinase A (PKA)-mediated beta lipid metabolism.

### Core gene families enriched in a specific pathogenic lifestyle

The network analysis also enabled the identification of abundant functional categories that contained functions enriched in the core of only one pathogenic lifestyle (Table 1, and corresponding detailed annotations in Additional file 2: Table S3).

Bcores – highly enriched functions were found mainly in: (i) nucleic acid metabolism; and (ii) signal transduction (GTPase, lysophospholipase, and tyrosine kinase activity). Less abundant specific Bcore-enriched functions included translation (t-RNA synthesis and ribosomal domain), and a KEGG ortholog with unknown function.

Hcores – highly enriched functions were found mainly in: (i) carbohydrate metabolism (certain glycoside hydrolase families, glucanosyltransferase, lactate dehydrogenase, expansin, and fucosidase); and (ii) amino acid metabolism (Gly, Ser, and His metabolism). Less abundant enriched Hcore functions included chaperones, CO_2_ sensing, rhodopsin-based light sensing, and 4 unknown function.

Ncores – highly enriched functions were found mainly in the transporters, and contained 2 domains with unknown functions. Less abundant annotations consisted of different proteases subfamilies in different pathogenic lifestyles (e.g., ubiquitin related-degradation in the Ncores).

### Identification of SSPs in core functions of pathogenic lifestyles

Predicted SSPs were found in 14% of the significantly enriched core functions (indicated in parentheses in Fig. 1, and in SSP column of Additional file 2: Table S3). In line with their role in pathogen–host interactions, most of the SSP functions were related to cutin and pectin degradation, cutinase, secondary metabolism, proteinaceous toxins, glycoside hydrolase, and signal transduction (tyrosine phosphatase activity) in the HN cores. In addition, complete genomic analysis (regardless of the network) showed that H contain significantly (40%) more predicted SSPs per genome than N, and pathogens have 66% more SSPs than saprophytes (*P* < 0.05, t-test).

### Evolutionary trajectory of fungal pathogens

**Fig. 3:**
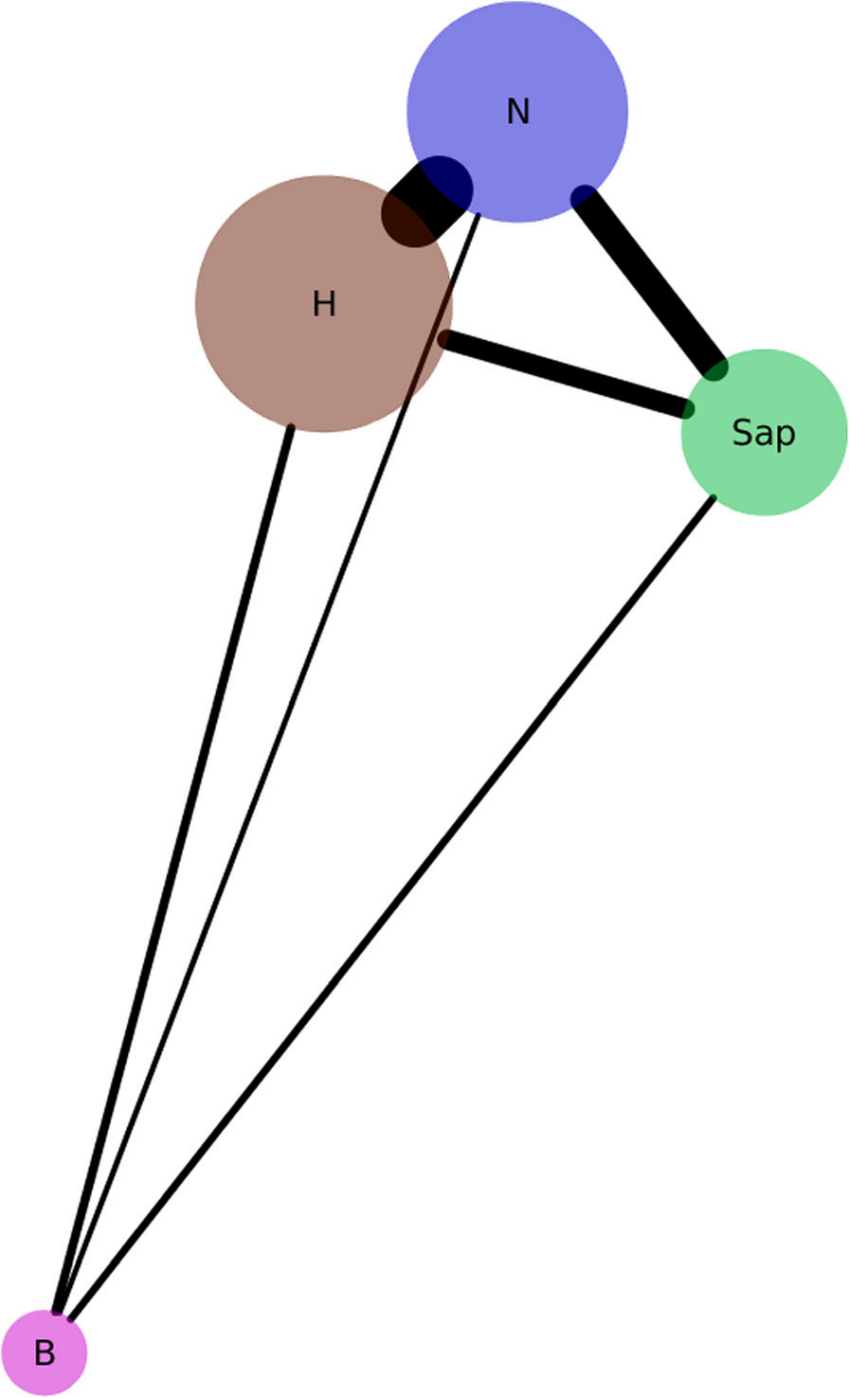
Presumed evolutionary trajectory of phytopathogenic and saprobic fungi illustrated by network of lifestyles’ shared functions. Edge thickness is in direct proportion to the count of shared gene families between different lifestyles (Table 2, see section “Gene families connecting a pair of lifestyles”, Methods), node size represents the average number of sequences per genome in a lifestyle. B – biotroph, H – hemibiotroph, N – necrotroph, Sap – saprotroph. Network image generated with Cytoscape version 3.3.0 [49] utilizing prefuse force directed layout algorithm

To study potential evolutionary trajectories of plant pathogens, we used a genomic approach to assess the number of gene families connecting a pair of lifestyles (Methods). This section encompassed all gene families (including core). Our results demonstrated (Fig. 3 and Table 2) a central place for N and H. Each of them shared the highest number of gene families with other groups. Accordingly, the highest number of gene families was shared between the N–H lifestyle pair, followed by N–Sap, H–Sap, H–B, and Sap–B.

**Table 2:** Counts of gene families (components) connecting a pair of lifestyles (see Methods). Related to Fig. 3. Numbers in parentheses are mean values obtained from 10,000 random simulations for each lifestyle. All simulations were found significant (*P* < 0.0001, non-parametric rank test, see Methods)

Lifestyle	Biotrophs	Hemibiotrophs	Necrotrophs	Saprotrophs
Biotrophs		360 (256)	261 (376)	292 (229)
Hemibiotrophs	233 (164)		2027 (708)	570 (418)
Necrotrophs	144 (207)	2020 (598)		846 (542)
Saprotrophs	228 (119)	701 (327)	957 (491)	

## Discussion

In this work, we focused on the core gene families that are predominant in the major lifestyles of filamentous fungal (and oomycete) plant pathogens. The network analysis used in our work illustrated that H has more significantly enriched core functions than N and B (in that order, Fig. 1). This is in line with the requirement of H to have both necrotrophic and biotrophic capabilities, in addition to functions associated with the transition between these lifestyles. This result is also in agreement with the higher number of SSPs per genome in H (regardless of core functions). The lowest number of biotrophic core functions can be explained by previous studies demonstrating that many functions which are required for virulence in this group have diversified, i.e., they are restricted to a specific taxonomic level or niche, and they are therefore not found in the core (see examples in [9, 22, 31, 33, 50]).

### Differentiating between lifestyles is empowered by core gene functions

One potential use of the core functions identified in this work was demonstrated by hierarchical clustering (Fig. 2). This analysis enabled differentiating B (together with some Sap genomes) from other pathogenic groups, obtaining most of the N and H genomes in HN clusters (2 with and 2 without Sap genomes), and obtaining a separate cluster of H. A comparative genomics study of 18 *Dothideomycetes* species (4 Sap, 5 N, 7 H, and 2 B), illustrated that clustering of these genomes using annotations of all genomic: (i) carbohydrate activity enzymes (CAZymes), showed 2 major clusters of HNSap and BHSap lifestyles; (ii) proteases, yielded mainly a separate H cluster, and a mixed HN cluster; (iii) lipases, showed mainly 2 HNSap clusters (the latter 2 contained also 21 outgroup genomes within Ascomycota and Basidiomycota). Thus, all genomic annotations of these 3 enzyme classes (CAZyme, proteases, and lipases) enabled obtaining similar (or less differentiating) separation between lifestyles compared to the use of selected core functions in the current work. All of the genomes with ambiguously characterized lifestyle (i.e., referred to as both H and N in the literature) were clustered with H (cluster 6, Fig. 2). Unfortunately, most of the work in the related literature does not include a detailed characterization or description of these lifestyles in each pathogen. However, as both necrotrophic and hemibiotrophic lifestyles are illustrated for a fungal pathogen in those studies, it is more likely to be hemibiotrophic, as its biotrophic stage could be more elusive (short or only appearing under specific conditions) and not identified in all studies. The distribution of saprophytes in biotrophic and necrotrophic lifestyles is in line with some studies suggesting that early diverging fungi were saprotrophic (see discussion below).

### Mapping core functions in pathogenic lifestyles

**Fig. 4:**
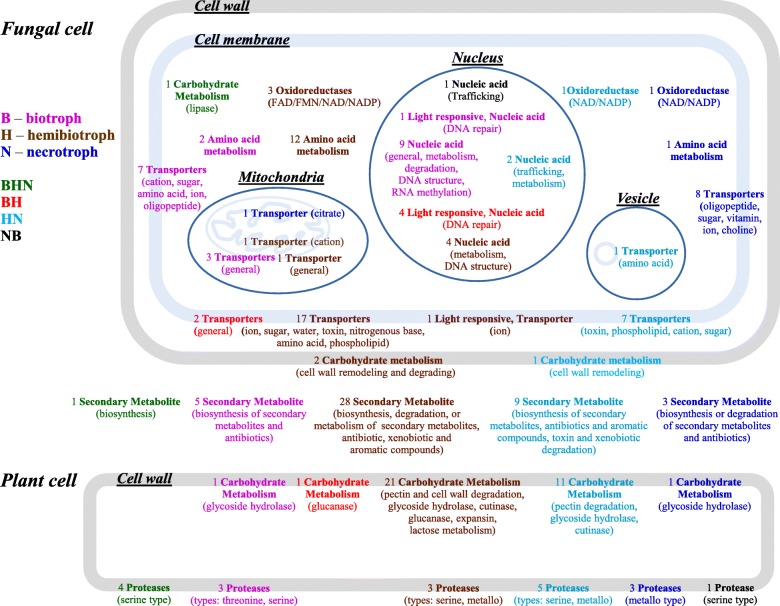
Map of significantly enriched core functions in different pathogenic lifestyles and their approximate subcellular location. Transporters are located on respective membranes, protease-and carbohydrate-associated functions are located on respective cell walls, and secondary metabolites are at the plant–pathogen interface (if subcellular location is not indicated, function is associated with the cytoplasm). The functions are colored based on their enrichment in a specific (e.g., purple for biotroph) or multiple (e.g., green for all three pathogenic lifestyles) lifestyle cores (see key on figure). Functional categories (Table 1) and their subcategories (Additional file 2: Table S3) are indicated by the following pattern: count functional category (subcategories), e.g., 3 proteases (type: serine, metallo) designating 3 enriched annotations in the Protease functional category, with serine peptidase and metallopeptidase subcategories

Our analysis provided a map of the core functions in the H, B, and N pathogenic lifestyles (Fig. 4, and Additional file 2: Table S3) derived from significantly enriched annotations in core gene families of these lifestyles.

### Functions enriched in all pathogenic lifestyles

Our analysis identified enrichment of members of serine protease family S8, and acyl-CoA oxidase in all three pathogenic lifestyles (indicated in green, BHN, Fig. 4). A previous computational study showed that the S8 serine proteases (subtilisin, identified in the BHNcore) are abundant across fungal lineages, and are highly correlated with pathogenic lifestyle in both animals and plants [5, 6]. A few studies illustrated the role of subtilisins (or subtilisin-like) in virulence, mediated mainly by cuticle degradation in fungal pathogens of insects (e.g., [51, 52]). Acyl-CoA oxidase (identified in the BHNcore) mediates the first step of beta oxidation which may be invoked by PKA, contributing to the pathogenicity process of phytopathogenic fungi [16]. The acetyl-CoA product of beta oxidation could enter the citric acid cycle to produce energy; alternatively, it is known to participate in the formation of metabolites such as glycerol, melanin, and glucose (via gluconeogenesis), known to contribute to virulence processes such as appressorium-mediated plant infection, in phytopathogenic fungi [53–56].

### The necrotrophic lifestyle

This section refers to fungal pathogenic functions that were enriched in the Ncore or in both N and Hcores, the latter attributed to the necrotrophic stage of H (indicated in blue, N; and in light blue, HN; Fig. 4). Our analysis revealed that the Ncore is enriched in functions associated with cell wall-associated degrading enzymes (e.g., pectinase, cutinase, and glycoside hydrolase family 28), toxin metabolism, proteases, and transport. These functions are probably needed to support necrotrophic growth, involving maceration of the host cell barriers (e.g., cell wall), and induction of host cell death followed by sequestering of nutritional compounds (e.g., amino acids and carbohydrates). For example, comparative analysis of mostly necrotrophic *Botrytis* species highlighted multiple cell wall- (carbohydrate-) degrading enzymes such as pectinases [57]. Toxin synthesis and degradation were abundant in the HNcore functions; these are known to mediate plant cell death and protection against plant defense mechanisms in the necrotrophic process [21, 24, 34, 35]. Accordingly, toxin transport was found to be enriched in these cores, in agreement with the previously identified arsenal of toxins in necrotrophs that mediate killing of the host cell prior to feeding on it [21, 34, 35]. Import of other compounds enriched in N (e.g., phospholipid and choline) could further support nutrition of the pathogen during the course of infection. Serine and metalloproteases: Roles in nutrient acquisition, host cell degradation and host-fungus interactions (e.g., neutralization of defense mechanism) have been previously illustrated for proteases in general [58, 59], and for serine proteases in particular [5, 60]. A computational study of serine proteases (found in the MEROPS database) illustrated that families S9 and S10 are abundant in fungal genomes, partially supporting their identification in the HNcore in our study; however, no correlation with pathogenic lifestyles was found for these families [5]. Comparative analysis of mostly necrotrophic *Botrytis* species facilitated the identification of a clade of 8 species with shared proteases (1 serine-type peptidase, 1 hydrolase acting on glycosyl bonds, 1 asparaginase, and 1 G1 endopeptidase) [57]. Pathogen proteases can participate in inhibiting plant defense components such as pathogenesis-related proteins (e.g., antimicrobial chitinases), and β-1,3-glucanases which mediate fungal cell wall hydrolysis (e.g., [61–63]). Metalloprotease activity (identified as enriched in Ncores) has been previously correlated with fungal phytopathogenic activity, directed mainly against plant chitinase used for defense [59]. One example is the FoMep1 protease secreted by *Fusarium oxysporum* f. sp. *lycopersici* which (together with a serine protease) was responsible for the degradation of chitinases of tomato [64]. To the best of our knowledge, the role of metalloprotease family M28, identified as enriched in HNcores in the current study, in fungal virulence against plants has not been previously demonstrated.

### The biotrophic lifestyle

This section refers to fungal pathogenic functions enriched in Bcores or in both B and Hcores, as the latter are attributed to the biotrophic stage of H (indicated in pink, B; and in red, BH; Fig. 4). The abundance of enriched functions related to signal-transduction processes (e.g., GTPase, lysophospholipase, and tyrosine phosphatase), and nucleotide metabolism (in specific DNA/RNA structures and recognition) could facilitate the tight regulation required for biotrophs to control their avoidance of plant defense mechanisms while feeding on the host compounds. The effect of Bcore functions of suppression of the plant defense system was demonstrated by a secreted tyrosine phosphatase of the bacteria *Pseudomonas syringae* that suppressed the immune responses of *Arabidopsis* by dephosphorylating a plant pattern recognition receptor [65]. Some of the small G-protein family of GTPases, such as Rac, Rho, and Rab, participate in regulating the mitogen-activated protein (MAP) kinase cascade in eukaryotes [66], which plays an important role in environmental sensing and consequent morphogenesis in phytopathogenic fungi [67, 68]. An example is the CDC42 Rho GTPase, which is involved in vegetative differentiation and is required for pathogenicity in the biotrophic wheat pathogen *Claviceps purpurea* [69]. A few functions associated with carbohydrate metabolism and secondary metabolism functions were enriched in the Bcore (Fig. 4, and Additional file 2: Table S3). This is in line with the comparative analysis of 4 downy mildew species and 3 *Phytophthora* species that also identified a few functions related to carbohydrates (such as pectin lyase and cutinase) and secondary metabolism (e.g., necrosis-inducing proteins) [70]). Comparative genomic analysis of various powdery mildew-causing pathogens also illustrated a reduced set of carbohydrate active enzymes devoted to plant cell wall depolymerization and secondary metabolites [12, 71]. Nucleotide metabolism: The potential role of RNA metabolism in the Bcore is supported by a recent study of the biotrophic obligate fungal pathogen *Plasmopara viticola*, which identified positive selective pressure (indicated by pairwise dN/dS values) in genes coding for RNA modification and processing [72]. One of these genes was the DEAD box helicase [72] (involved in transcription, splicing, and RNA transport), observed in the Bcore, which is known to regulate multiple virulence genes in the fungal pathogen of mammals, *Cryptococcus neoformans* [73]. Analysis of genes under positive selection in the biotroph *Plasmopara viticola* also highlighted genes associated with RNA metabolism, mRNA maturation and processing, or rRNA and tRNA modification, and DEAD/DEAH RNA helicase [72]. Specific histone residues are known to undergo posttranslational modification (mainly methylation and acetylation) [74], and therefore Bcore histone variants could affect histone modification, which might ultimately affect transcription and epigenetic-based regulation. The role of histone modification (i.e., methylation and acetylation) has been demonstrated in the pathogenicity process of several phytopathogenic fungi [75, 76]. For example, deletion of *gcn*5 histone acetyltransferase in the biotrophic fungal pathogen *Ustilago maydis* significantly reduced the infection process on maize [77].

### Cryptochromes and photolyases in the Bcore

Cryptochromes are photoreceptors that are closely related to photolyases, but they do not necessarily exhibit DNA-repair functionality and may possess regulatory functions [78]. In the biotrophic fungal pathogen *Blumeria graminis* f. sp. *hordei*, UV-C irradiation inhibited conidial germination and appressorium formation (participating in host penetration), while upregulation of 3 putative photolyases was observed, suggesting their potential role in protection from UV-C [79]. Disruption of PHL1 (a cryptochrome/photolyase homolog) in the hemibiotrophic phytopathogenic fungus *Cercospora zeae-maydis* inhibited light-dependent DNA repair (photoreactivation) activity, and exhibited reduced expression of another cryptochrome, and of genes involved in nucleotide excision and chromatin remodeling during DNA-damage repair [80, 81]. Although cryptochromes were not enriched in the Ncore, they are found in several N genomes. An interesting example is the necrotrophic fungal pathogen *B. cinerea*, where the chryptochrome BcCRY1 acts as the major photolyase in photoprotection, and the cryptochrome BcCRY2 participates in regulating photomorphogenesis (repression of conidiation) [82]. Although this function, may appear in a different path in biotrophs, it could play an important role in fungal plant pathogens. These findings, together with their position in the Bcores, suggest that cryptochromes mediated photoprotection, and photomorphogenesis could play a central role in the biotrophic lifestyle.

### The hemibiotrophic lifestyle

One intriguing role for functions enriched only in the Hcores (indicated in brown, Fig. 4) might be participation in the shift between lifestyles. Degradation of lignocellulose compounds: While some GH families identified in the Hcore are active on a narrow range of substrates (e.g., xylanase for GH12 and galactanase for GH53), others (e.g., GH 1, 3, and 11) have diverse activities [83, 84]. Comparative analysis of plant cell wall-degrading enzymes in fungal genomes also showed that the GH3 family is significantly more abundant in hemibiotrophs (and in necrotrophs) than in biotrophs [7]. Lactate dehydrogenase (identified in the Hcore) may support pyruvate production, during infection, from plant-based lactate, generated as a byproduct of plant primary metabolism [85], and the resulting pyruvate could support energy needs of the infection. The observed differences between the lifestyles in a profile of carbohydrate metabolism-related functions could be the result of adaptation of fungal pathogens to different plant biomass (e.g., composition of plant cell walls affecting penetration). Alternatively, different profiles of these functions could generate changes in environmental conditions (e.g., changes in the composition of soluble compounds or pH) that would serve as a cue for related functions, such as transition between lifestyles. It is known, for example, from several phytopathogenic fungal systems that favorable pH conditions promote the infection process in the necrotrophic stage (e.g., in *Sclerotinia sclerotiorum* [86] or *C. gloeosporioides* [38, 87]). Expansins are cell wall-loosening proteins that are abundant in plant-associated microbes, including plant pathogens (according to a genomic search in NR, NCBI [88]). A few studies have explored the role of expansins in phytopathogen virulence [89, 90]. In the hemibiotrophic cacao pathogen *Moniliophthora perniciosa*, aggregated MpCP2 with cellulose-loosening activity was shown to promote spore (basidiospore) germination and subsequent tube growth, whereas the MpCP2-encoding gene was expressed in necrotic seeds; thus, MpCP2 had a potential role in both biotrophic (spore germination) and necrotrophic (seed) stages [89]. Despite the observed abundance of expansin in plant pathogens, there are very few genetic studies suggesting a potential role for fungal expansins in the virulence of phytopathogenic fungi. Thus, the current work, highlighting its position in the Hcore, suggests that functional studies of expansins’ involvement in virulence are likely to be fruitful.

### Light and CO_2_ perception in the Hcores

Carbonic anhydrase facilitates CO_2_ sensing and subsequent differentiation, and virulence in the two human pathogens *Candida albicans* and *C. neoformans* [91–93]. These studies, together with its enrichment in the Hcores, suggest a similar role in sensing alterations in CO_2_ level during plant infection, followed by induction of processes such as the transition between lifestyles. Rhodopsins: Fungi contain bacteriorhodopsins/microbial opsins that are light-driven ion pumps generating proton gradients across membranes [94]. Infection of rice plants with the rhodopsin-deficient mutant homolog (CarO) of *Fusarium fujikuroi* (ambiguously referred to as H or N, see Additional file 2: Table S1) showed more severe symptoms than the control strain, indicating a potential role of rhodopsin in the regulation of plant infection [95]. Silencing of the opsin ortholog Sop1 in the necrotroph *Sclerotinia sclerotiorum* resulted in reduced necrotic growth on oilseed rape leaves, and higher sensitivity to osmotic stress [96]. This illustrates the role of rhodopsins in light sensing and photomorphogenesis of phytopathogenic fungi, and along with its identification in the Hcores, suggests that alterations in light regime could play a role in virulence functions of hemibiotrophs, such as transitioning between lifestyles.

### Evolutionary trajectory of fungal pathogens

Early diverging fungal lineages (e.g., Blastocladiomycota and Chytridiomycota) identified in phylogenetic studies [4, 97–100] contain mainly saprobes and obligate biotrophs (and some endosymbionts) [98, 101]. Our analysis complements these observations by suggesting scenarios that presumably followed the emergence of these two lifestyles. Primordial fungal saprobes, able to both decompose organic compounds and degrade debris of ancestral plants, presumably evolved a necrotrophic lifestyle as suggested by the highest number of shared gene families between Sap and N (Fig. 3). Although an alternative route could be suggested from the high number of Sap families shared with H, the evolution into N supplies a simpler explanation, which could have been followed by the subsequent emergence of H. The necrotrophic lifestyle could have initially evolved by acquisition of a relatively small number of toxins and lytic enzymes able to cause cell death. In this regard, the study of shared pectinase families in Dikarya and early diverging *Gonapodya prolifera*, a saprobe (member of the Chytridiomycota) able to grow on pectin as a carbon source, provides evidence for a common fungal ancestor able to feed on ancestral plant/algal pectin-containing debris [97]. Alternatively, or simultaneously, a primordial B could have acquired necrotrophic mechanisms, shifting to a hemibiotrophic lifestyle as illustrated by the highest number of gene families shared by B and H; subsequent loss of functions could have generated the necrotrophic-only lifestyle. The initial step of this scenario, starting with an ancestral biotrophic lifestyle, is more complicated than the aforementioned saprobic origin, as it requires acquisition of functions regulating the hemibiotrophic shift in addition to necrotrophic functions. However, it is supported by phylogenetic studies which have identified an early diverging sister clade of fungi (the Cryptomycota and Microsporidia taxa) that is made up of obligate biotrophic endoparasites [98, 100]. In both scenarios, acquiring a new lifestyle would have been advantageous in competition for niche/food resources.

## Conclusions

Our network analysis provides a map of the core functions in three major lifestyles of phytopathogenic fungi and oomycetes. The core functions highlighted in this work, which have not been previously associated with studied pathogenic lifestyles, including several enriched orthologs or domains with unknown function and some core families that cannot be annotated (Additional file 2: Table S2), open new avenues for future research that will enable a better understanding of these pathogens, and the discovery of novel functions associated with pathogenicity. It would make sense to start with core families with unknown function that contain SSPs, as the latter are often associated with pathogenicity. Regulatory mechanisms found in the Hcore functions include light- and CO_2_-sensing functions that could mediate important roles in this group, such as transition between lifestyles. These roles could also be regulated by changes in environmental composition resulting from the different core of lignocellulose-degrading enzymes found in this lifestyle. The presence of photoreceptors (cryptochrome and rhodopsin) in the cores of plant pathogens raises the novel possibility of their central role in virulence, which is in agreement with the understanding that FEPs coevolved with photoautotrophic plant hosts. Our finding of light-sensing functions in the pathogen cores is partially supported by a survey of 22 Ascomycota which showed that they contain light-sensing mechanisms. These should confer better adaptation (protection, phototropism, morphogenesis, and circadian clock activity) under different light regimes [94].

The selected set of enriched core functions identified in our work can be used in other studies and applications. For example, these core functions can assist in identifying the pathogenic potential of samples analyzed by metagenomics or single-cell genomics. An interesting application in this direction would be to facilitate advanced agrotechnical practice, which is based on soil and leaf metagenomics (in addition to chemical monitoring) in future “next generation agriculture” [102, 103]. Last, empowered by the whole genomic network methodology, our analysis offers potential evolutionary scenarios following the emergence of an early branching saprobe and/or the obligate biotroph described in previous works.

## Methods

### Selected organisms

The data sets analysed in this study (downloaded at February 2016) can be found mainly in the National Center for Biotechnology Information, and in the Ensembl genomes databases using the accession numbers (and links) listed in Additional file 2: Table S1. The 82 selected genomes fungi and oomycetes represent the following lifestyles: 18 B, 20 H, and 22 N, 17 Sap (control or non-pathogens), and 5 pathogens ambiguously annotated as N or H (Additional file 2: Table S1). All biotrophs were treated uniformly in downstream analyses. The lifestyle of an organism was determined from either the respective database from which the sequences were collected, or the literature.

### Construction of the pan-proteome network

The pan-proteome sequence-similarity network was computed using EGN [104] for the 82 genomes with their 1,041,984 predicted protein sequences (hereafter, protein sequences) aligned using all-vs.-all BLASTP. Each node in the network represents a protein sequence from the 82 proteomes, and edges represent sequence similarity between pairs of protein sequences above a selected threshold that is accepted in the field [48] with minor modifications: minimal sequence length of 40 residues, *E* value < 10^− 4^, sequence identity ≥35% and minimal match coverage ≥70%. Only subgraphs with ≥5 nodes were included in further analyses (covering 30% of the subgraphs, and 85% of the sequences, Additional file 1: Figure S2). The resulting network contained a large number of separate (unconnected) subgraphs (referred to as components), representing an operational gene family (referred as, gene family) whose sequences do not share significant similarity with other components [48]. When stringent criteria (larger thresholds) are enforced, most of these families putatively have the same or a closely related major function. Our pan-proteome network consisted of approximately 53 K gene families comprising 704 K protein-encoding genes (Additional file 1: Figure S2). Most (94%) of these were relatively small families (5 to 82 proteins per family, containing 1 protein per organism on average), that together comprised 61% of the sequences.

Python scripts were used to identify components of interest (e.g., having selected lifestyles), and to calculate relevant features (e.g., number of nodes per lifestyle, edges per each pair of lifestyles, or edges connecting selected lifestyles).

### Core components (i.e., core gene families)

In this work, we defined a core component of a lifestyle as a component containing proteins derived from ≥70% of the organisms of that lifestyle. For example, a core component of N will have proteins from at least 70% of the 22 N used to construct the network (Additional file 2: Table S1). A Venn diagram was used to demonstrate the uniqueness of the core components (Additional file 1: Figure S1) using the web tool InteractiVenn [105].

### Annotation of protein-encoding genes

All the 1,041,984 protein sequences of all the 82 organisms (genomes) were annotated based on several established platforms (Additional file 2: Table S2). KEGG orthologs and related pathways were identified using web-based KEGG Automatic Annotation Server (KASS [106]) based on BLAST single-directional best hit (SBH) against a selected list of genomes (Additional file 2: Table S4) using default thresholds and parameters. Protein domains were searched with standalone InterProScan [107] against 6 databases (Pfam, ProDom, Gene3D, TIGRFAM, ProSitePatterns, and PRINTS; recommended by InterPro, personal communication) using default thresholds and parameters. Protease families were identified using BLASTP (*E* value < 0.001) against the MEROPS database [108] downloaded in November 2017. Carbohydrate-degrading enzymes were also identified using a Hidden Markov Model (HMM) search against the CAZymes HMM database (dbCAN-fam-HMM) [109], downloaded in October 2017, using protein sequences (with default steps and parameters as suggested for fungi by the developers). However, functions found by this database (which were significantly enriched, see below) were also found by other approaches (e.g., KEGG orthologs, and InterPro domain search), and therefore they were not included in this final report. Possible secreted effectors were predicted by choosing proteins with sequence length ≤ 300 residues [9, 110], at least 2% Cysteines in the protein sequence (for short proteins, at least 2 Cys) [111], signal peptide based on standalone SignalP 4.1 [112], and no transmembrane region based on standalone TMHMM [113].

### Calculation of enrichment and significance of core pathogenic functions

Enrichment of an annotation (i.e., KEGG, IPR, MEROPS, CAZyme) in the core components was calculated using the following equation (CPSA_SPC/CPAA_SPC)/(CPSA_B/CPAA_B) [48, 114], where CPSA_SPC stands for Count of Proteins with Specific Annotation ID in Selected Pathogenic Core (SPC), CPAA_SPC stands for Count of Proteins with Any Annotation ID in SPC, CPSA_B stands for Count of Proteins with Specific Annotation ID in Background, CPAA_B stands for Count of Proteins with Any Annotation ID in Background. We calculated enrichment for several combinations of Background and SPC: (i) general background comparing selected core to the entire network. Background - entire network excluding SPC; SPC - core components of selected lifestyle. For example (Proteins with selected KEGG ID in Ncore/Proteins with Any KEGG ID in Ncore)/(Proteins with selected KEGG ID in the rest of the network/Proteins with Any KEGG ID in the rest of the network); (ii) to validate enrichment relative to non-pathogens, we compared the core of a selected pathogenic lifestyle to the non-pathogenic lifestyle of Sap. Background – Sap in core components of selected pathogenic lifestyle; SPC - selected pathogenic lifestyle in core components of selected lifestyle. For example - (Proteins with selected KEGG ID in N organisms of Ncore/Proteins with Any KEGG ID in N organisms of Ncore)/(Proteins with selected KEGG ID in Sap organisms of Ncore/Proteins with Any KEGG ID in Sap organisms of Ncore). In addition we have validated that none of the significantly enriched functions identified in the pathogenic cores were significantly enriched in the non-pathogenic Sap cores (e.g., none of the KEGG IDs enriched in Ncore were enriched in the Sapcore); (iii) to further confirm that the functions of a selected pathogenic lifestyle are not highly abundant in the non-pathogenic Sap lifestyle (i.e., missed in calculations above because they were omitted from the network due to the thresholds selected to generate edges), we also tested the organisms’ background regardless of the network. Background – proteins from Sap; SPC – all annotated proteins from the selected lifestyle. For example (Proteins with selected KEGG ID in N/Proteins with Any KEGG ID in N)/(Proteins with selected KEGG ID in Sap/Proteins with Any KEGG ID in Sap).

Significance of each annotation (i.e., KEGG, IPR, MEROPS, CAZyme, Fig. 1, Table 1 and Additional file 2: Table S3) was calculated using Fisher’s exact test in the scipy module of python [114]; only annotations with *P* < 0.05 and enrichment > 1.1 in all combinations of Background and SPC (above) were considered significantly enriched, and used in further analyses. The core functions were hierarchically clustered (Fig. 2) based on their abundance in each of the 82 organisms with the hclust package in R based on Euclidian distance using Ward’s hierarchical agglomerative clustering [115].

### Gene families connecting a pair of lifestyles

Gene families connecting a pair of lifestyles were defined as components (i.e., operational gene families, see above) containing at least 1 direct connection (network edge) between proteins of 2 lifestyles (other lifestyles may exist in that component), and that the lifestyle selected as the focus of the analysis (see random simulated lifestyles below) does not have a direct connection with any other lifestyle. For example, for the B–N connection, such a component would contain at least 1 network edge connecting proteins from these two lifestyles, and if the B lifestyle is at the focus of the analysis, then it will not be connected to any other lifestyle (i.e., only B–N, and B–B edges are allowed for B). In contrast to the analysis described in previous section which is aimed to identify core functions of fungal and oomycete pathogens by utilizing genome sequences of representatives from both of these groups, the analysis described here is focused on potential evolutionary connections between lifestyles (i.e., B, N, H, and Sap). However, taxonomic classification and phylogenetic analyses have suggested that oomycetes form a clade that is distinct from fungi [97, 98]. To prevent possible mix between clade (i.e., oomycetes and fungi) and lifestyle connections in the evolutionary analysis we have excluded the oomycetes from this analysis.

### Significance of counts of gene families connecting a pair of lifestyles

To assess the significance of counts of gene families connecting a pair of lifestyles, we used random simulated lifestyles, in which labels (i.e., the lifestyle) for the organisms were randomly shuffled while preserving the numbers of genomes belonging to each lifestyle. In Table 2, the lifestyle in columns (the lifestyle being focused on) was kept intact, whereas lifestyles in rows were randomly shuffled. Once a new label was attributed to a genome, all of its proteins (nodes) were labeled as this new lifestyle. For each lifestyle, numbers of gene families connecting a pair of lifestyles were enumerated. The process was repeated 4 × 10,000 times for each focused-on lifestyle (Table 2). All simulated counts were averaged and are reported in parentheses in Table 2. Significance of the values was computed using non-parametric, empirical *P*-values, based on ranked real values of the simulations. When the real value was higher than 1 out of 10,000 simulated values, we attributed a *P*-value of < 0.0001. Normalized values were also significant for most cases (Additional file 2: Table S5), although they contained few non-significant differences between real data and simulations (indicated by asterisks in Additional file 2: Table S5), it did not affect the results. In the other cases, there were significantly more or less exclusive gene families between two groups in the real data than in the simulations. Normalization was generated by dividing the count of the components of interest by the maximum number of components that can exist in a studied network; e.g., if there are 15 K components of N, and 10 K components of B, the maximum possible number of components with an N–B connection (network edge) is 10 K. Based on the counts of gene families connecting a pair of lifestyles, a network was generated (Fig. 3), using Cytoscape version 3.3.0 [49].

Interpretation of the simulations. For a pair of column X and row Y that shows more gene families than in the random simulation (e.g., column B and row H), genomes or gene families from lifestyle X are more similar to genomes or gene families from lifestyle Y than to genomes or gene families from any other lifestyle (in the dataset). Accordingly, when there are significantly less gene families in the real data than in the simulations (e.g., column B and row N), genomes or gene families in lifestyle X are more dissimilar to genomes or gene families in lifestyle Y than to genomes or gene families from any other lifestyle (in the dataset).

## Supplementary information

**Additional file 1 : Figure S1**. Distribution of core components among all four lifestyles. B, H, N and Sap stands for Biotroph, Hemibiotroph, Necrotroph, and Saprotrophs respectively. **Figure S2.** Cumulative frequency of components and sequences in the network.

**Additional file 2 : Table S1.** Organisms used in this study. **Table S2.** Frequency of annotated core families and their proteins (see sections “Core components” and “Construction of the pan-proteome network”, Methods). **Table S3.** Significantly enriched annotations in core components of Biotroph (B), Hemibiotroph (H) and Necrotroph (N). * Annotations starting with: K, KEGG ortholog; IPR, InterPro domain; MER, MEROPS proteases. ** Functional categories: N, nucleic acid related; L, light responsive; A, amino acid metabolism; T, transporters; C, carbohydrate metabolism; P, protease and peptidases; SM, secondary metabolites (including toxins); O, oxidoreductases (with no other function); Tf, trafficking; E, energy; ST, signal transduction. *** Following procedure described in methods, and subsequent manual curation. **Table S4.** Selected organisms used in KAAS analysis for identififcation of KEGG orthologs. **Table S5.** Normalized counts of gene families (components) connecting a pair of lifestyles. Numbers in parenthesis are the normalized mean values obtained from random 10,000 simulations for each lifestyle. A single asterisk indicates nonsignificant values (*P* > 0.01, non-parametric rank test, see Methods) (XLS 3237 kb)

## Data Availability

The data sets analysed in this study can be found in The National Center for Biotechnology Information Taxonomy database using the accession numbers listed in Additional file 2: Table S1.

## Abbreviations

B: biotroph
CAZyme: carbohydrate-active enzymes
FEP: filamentous eukaryotic pathogen
H: hemibiotroph
HMM: hidden Markov Model
KEGG: Kyoto Encyclopedia of Genes and Genomes
MAPK: mitogen-activated protein kinase
N: necrotroph
PKA: protein kinase A
SPC: selected pathogenic core, SSP – small secreted protein
