# Supplementary figures and images for: Network analysis exposes core functions in major lifestyles of fungal and oomycete plant pathogens
Source: BMC Genomics. 2019 Dec 26;20:1020. doi: 10.1186/s12864-019-6409-3 (PMC6933724; doi:10.1186/s12864-019-6409-3)

Figure S1

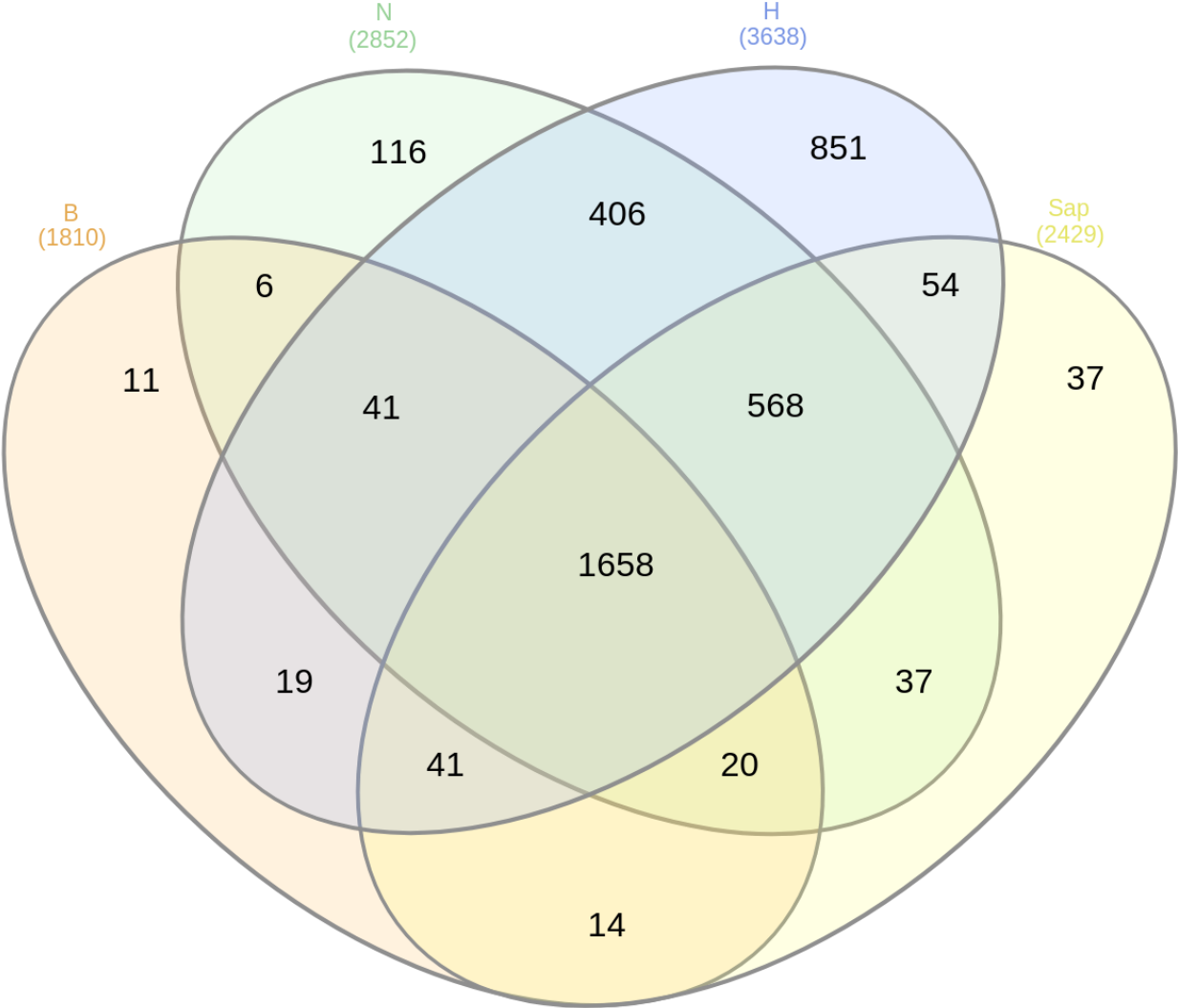

Figure S2

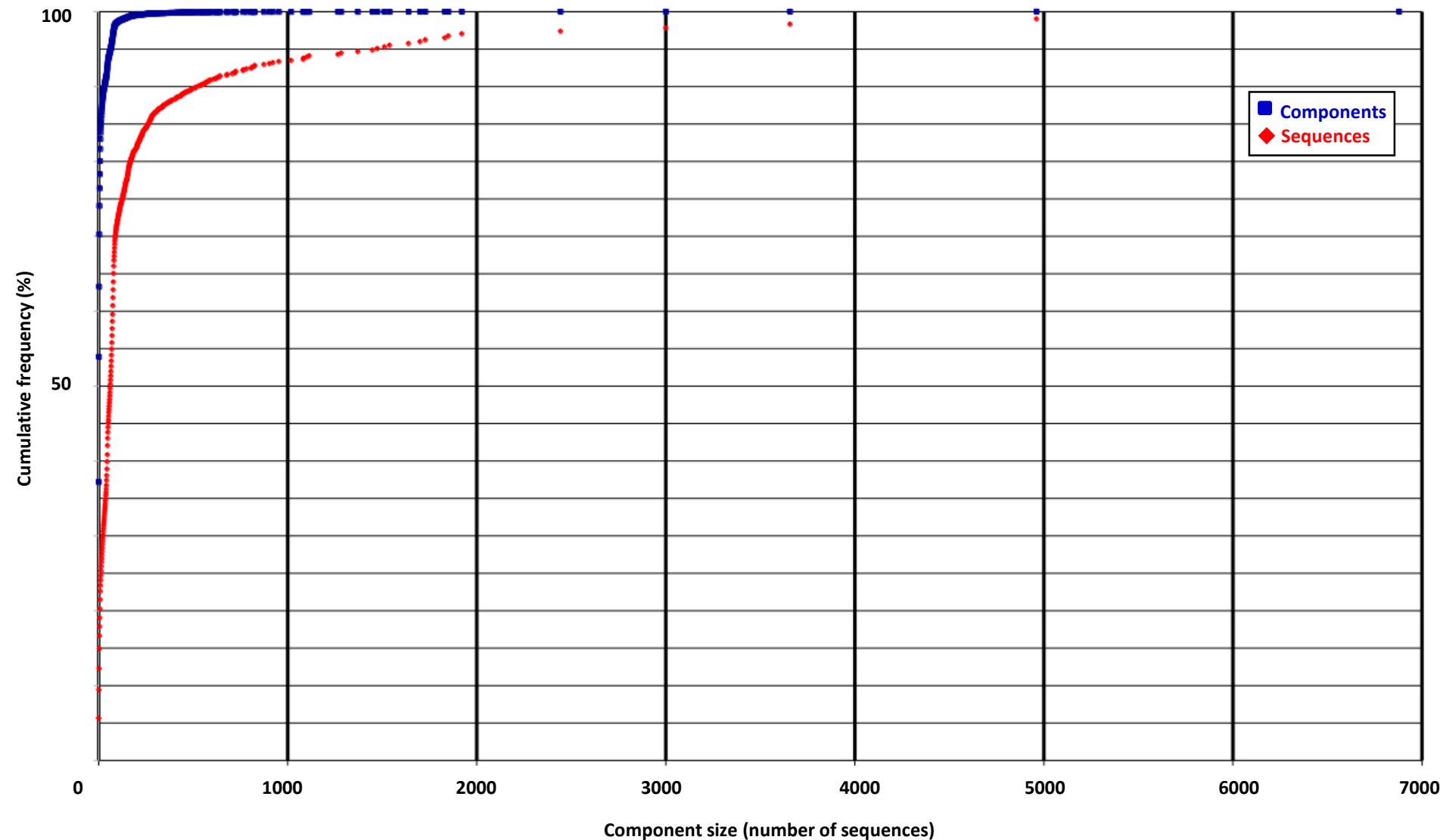

Supplement: Supplementary file 1 — Additional file 1 : Figure S1. Distribution of core components among all four lifestyles. B, H, N and Sap stands for Biotroph, Hemibiotroph, Necrotroph, and Saprotrophs respectively. Figure S2. Cumulative frequency of components and sequences in the network. [file 12864_2019_6409_MOESM1_ESM.pdf]
